# A parsimonious model to validate cost-effectiveness analyses on preventive health care

**DOI:** 10.1186/s12913-021-07217-2

**Published:** 2021-11-09

**Authors:** Afschin Gandjour

**Affiliations:** Frankfurt School of Finance & Management, Adickesallee 32-34, 60322 Frankfurt am Main, Germany

**Keywords:** Cost-effectiveness analysis, Model, Validation

## Abstract

**Background:**

The effect of preventive health care on health expenditures is ambiguous. On the one hand, prevention reduces the costs of future morbidity. On the other hand, prevention leads to costs of life extension. The purpose of this paper is to develop a parsimonious model that determines for a preventive measure of interest whether savings from preventing morbidity are more than offset by the costs of living longer, resulting in a net expenditure increase.

**Methods:**

A theoretical model was built based on a Weibull survival function. It includes savings and life extension costs over the remaining lifetime. The model was applied to the example of obesity prevention.

**Results:**

The model shows that the cost consequences of prevention are essentially driven by two factors: i) the relative reduction of morbidity-related costs, which determines the amount of savings from avoiding morbidity; and ii) the hazard ratio of death, which determines the amount of life extension costs. In the application example, the model is able to validate the results of a more complex cost-effectiveness model on obesity prevention.

**Conclusions:**

This work provides new insight into the lifetime cost consequences of prevention. The model can be used both to check plausibility of the results of other models and to conduct an independent analysis.

Under a budget constraint publicly financed preventive health care is pressurized to demonstrate savings. While prevention reduces the costs of future morbidity, it increases the probability that new and costly diseases such as Alzheimer disease and cancer occur during the additional lifetime. Prolonging life can also extend the period of existing disease, thus resulting in continuous disease-related expenditures.

In general, three categories of prevention exist: primary, secondary, and tertiary [1]. Primary prevention keeps the disease from occurring at all by removing its causes. Secondary prevention detects early disease when it is asymptomatic and when treatment can stop it from progressing. And tertiary prevention refers to those clinical activities that prevent further deterioration or reduce complications after a disease has declared itself [1]. In reference to the example of obesity, primary prevention intends to prevent the incidence of overweight/obesity. Secondary obesity prevention intends to help overweight and obese individuals obtain a healthy weight. And tertiary prevention of obesity aims at resolving weight-related comorbidities or at least decreasing their severity [2].

Given that successful primary obesity prevention prevents obesity-related disorders such as type 2 diabetes as a consequence of reducing the incidence of overweight/obesity, it is predicted to increase life expectancy, bearing in mind that evidence for the effectiveness of policies to prevent obesity still remains limited [3]. In the case of primary prevention of obesity, the relevant question, under a budget constraint, thus becomes whether expenditures during additional lifetime more than offset savings from preventing the incidence of obesity.

When avoiding obesity-related disorders leads to smaller life extension costs than savings, normal-weight people have lower costs over remaining lifetime compared with obese persons and vice versa [4]. Modelling studies based on U.S. data suggest that expenditures over remaining lifetime are projected to drop by preventing obesity in the general population at age 20 to 70 [5–8]. On the other hand, a Dutch modelling study [9] shows an increase in expenditures over remaining lifetime by 13% when preventing obesity at age 20. Among the U.S. modelling studies, a study published by Allison et al. [5] is perhaps most similar to that of van Baal et al. [6]. It also uses secondary data and simulates obesity prevention at the age of 20 years. As both studies consider costs unrelated to obesity-related disorders in added life years, the latter cannot explain diverging results between the two studies. Similarly, a decrease in obesity-related mortality with age (in agreement with Flegal et al. [10]) is incorporated in both studies. Instead, age-specific differences in mortality and/or costs are likely to explain the differences in lifetime expenditures.

As seen in the example of obesity prevention, we may not be able to fully explain why different models arrive at different results.^1^ The purpose of this paper is to develop a parsimonious model that builds on the fundamental drivers of downstream health expenditures, i.e., savings from preventing morbidity and costs of living longer. The model is able to determine whether savings from preventing morbidity are more than offset by the costs of living longer, resulting in a net expenditure increase. The primary use for the model is to check the internal validity of other, more comprehensive models and explain their results. As a secondary purpose, the model can be used to predict costs and cost-effectiveness when no comprehensive model is available yet. In this paper it is applied to obesity prevention in order to serve both purposes.

## Model structure

From a payer or societal perspective costs stemming from a preventive program can be divided into the following components: (1) program-related costs which includes costs of screening, behavioral counseling, immunization, preventive medication, and treatment of side effects; (2) savings from avoided clinical events and reduced morbidity; and (3) life extension costs stemming from reduced mortality. While a payer perspective considers direct medical and non-medical costs, a societal perspective also captures indirect (non-medical) costs, i.e., productivity gains or losses. Productivity gains or losses can be considered part of the second and third cost component but can also be captured separately.

In the following, this study only considers direct health care costs, i.e., productivity gains from prevention are not taken into account. Furthermore, as this study focuses on downstream costs of prevention, it only includes savings from avoiding morbidity and expenditures on additional life years. This study models both cost components separately in order to keep the model transparent. Savings from reducing morbidity (∆*MC*, morbidity costs) yielded in period *j* as a result of prevention (p) are calculated by multiplying period-specific health care costs of survivors without prevention (*C*_*j*_) by the period-specific relative cost reduction of prevention:

1 $$ \Delta  {MC}_j^{\mathrm{p}}={s}_i\times \left(1-{d}_j\right)\times \left(1-{RMC}_j^{\mathrm{p}}\right)\times {C}_j, $$

where *s*_*i*_ is the probability of surviving from age *i* to *j*, *C*_*j*_ is the cost incurred during time interval (*j*, *j* + 1), *d*_*j*_ is the probability of death in period *j* without prevention, and ${RMC}_j^{\mathrm{p}}$ is the relative morbidity cost in period *j* as a result of prevention compared to no prevention. Note that the equation disregards the effect of prevention on mortality and the resulting costs. These effects are modelled separately below.

Cumulative savings over *n* time periods for an individual at age *i* = 1 are calculated by summing up period-specific savings. Formally:

2 $$ \Delta  {MC}^{\mathrm{p}}=\sum \limits_{j=1}^n\left(\prod \limits_{i=1}^j{s}_i\right)\times \left(1-{d}_j\right)\times \left(1-{RMC}_j^{\mathrm{p}}\right)\times {C}_j. $$

Note that if eq. 2 were applied over the remaining lifetime of an individual, it would assume that morbidity can be compressed indefinitely. Otherwise, the period of morbidity compression needs to be defined by adjusting the number of time periods *n*.

Now, I turn to the costs of life extension. They depend on the survival time gained through prevention, which itself depends on the hazard ratio of mortality and the time-dependence of survival without prevention. The latter, in turn, can be characterized by a Weibull survival model [11], which is the most widely used parametric survival model ([12], p. 272) and has favorable properties as detailed below. Its survival function is:

3 $$ s(t)={e}^{-\lambda {t}^{\alpha }}, $$

where *s*(*t*) is the probability of survival until time *t*, *λ* is the hazard rate, and *α* is a shape parameter. The shape parameter determines the shape of the hazard function, i.e., allows the hazard rate to increase or decrease monotonically with respect to time. If *α* > 1, then the hazard increases with time (for *α* = 2 the hazard increases linearly with time). If *α* = 1, then the hazard is constant and the Weibull model reduces to the exponential model. And if *α* < 1, then the hazard decreases with time ([12], p. 272).

An important feature of the Weibull model is its ability to accommodate an accelerated failure time (AFT) model. The Weibull AFT model has the essential property of a multiplicative effect with survival time ([12], p. 265). This multiplicative effect can be demonstrated by solving eq. 3 for *t*: ([12], p. 276).

4 $$ t={\left[-\ln s(t)\right]}^{1/\propto}\times \frac{1}{\lambda^{1/\propto .}}. $$

The equation shows that factor $\frac{1}{\lambda^{1/\propto }}$ scales survival time (*t*). If we insert the hazard rates of prevention and no prevention into the equation, then their ratio (hazard ratio) raised to the power 1/*α* is reciprocal to the relative increase in survival time due to prevention *t*^p^/*t*:

5 $$ \frac{t^{\mathrm{p}}}{t}=\frac{1}{{\left({\lambda}^{\mathrm{p}}/\lambda \right)}^{1/\propto }}. $$

In order to calculate life extension costs, we need to determine the absolute increase in survival time:

6 $$ {t}^{\mathrm{p}}-t=\frac{t^{\mathrm{p}}-t}{t}\times t=\left(\frac{t^{\mathrm{p}}}{t}-1\right)\times t=\left(\frac{1}{{\left({\lambda}^{\mathrm{p}}/\lambda \right)}^{1/\propto }}-1\right)\times t. $$

An increasing hazard rate (α > 1) as is typically seen beyond childhood thus lowers the gains in lifetime through prevention.

The inverse relationship between hazard ratio and survival time is used to model costs from life extension in the following. To this end, consider that life extension costs are a product of the additional survival time and the average cost per unit of time. Additional survival time, in turn, can be expressed as a function of the survival time without prevention based on eq. 6. In addition, costs per unit of time need to be adjusted for the morbidity reduction through prevention. Based on these considerations costs of extending a single period *j* ($\Delta  {LEC}_j^{\mathrm{p}}$) are calculated as follows:

7 $$ \Delta  {LEC}_j^{\mathrm{p}}=\left(1/{HR}_j^{1/\propto }-1\right)\times {s}_i\times \left(1-{d}_j\right)\times {C}_j\times {RMC}_j^{\mathrm{p}}, $$

where *HR* is the hazard ratio of prevention. Note that eq. 7 excludes *t* because it refers to a single period (with a standardized length of 1) and hence considers expansion of this period only.^2^ Furthermore, if *HR* refers to overall survival, life extension costs include medical costs unrelated to the prevented disease.

The sum over all periods then yields total life extension costs:

8 $$ \Delta  {LEC}^{\mathrm{p}}=\sum \limits_{j=1}^n\left(\prod \limits_{i=1}^j{s}_i\right)\times \left(\frac{1}{HR_j^{\frac{1}{\propto }}}-1\right)\times \left(1-{d}_j\right)\times {RMC}_j^{\mathrm{p}}\times {C}_j. $$

In the following, I derive the general condition under which savings are exactly offset by life extension costs. This enables us to predict for specific prevention examples whether savings from reducing morbidity are more than offset by the costs of living longer. To this end, I equate eqs. 2 and 8:

9 $$ \sum \limits_{j=1}^n\left(\prod \limits_{i=1}^j{s}_i\right)\times \left(1-{d}_j\right)\times \left(1-{RMC}_j^{\mathrm{p}}\right)\times {C}_j=\sum \limits_{j=1}^n\left(\prod \limits_{i=1}^j{s}_i\right)\times \left(\frac{1}{HR_j^{\frac{1}{\propto }}}-1\right)\times \left(1-{d}_j\right)\times {RMC}_j^{\mathrm{p}}\times {C}_j. $$

It is easy to see that several factors on both sides of the equation cancel out. For the same reason an increase in period-specific health care costs of survivors (${C}_{\overline{\mathrm{p}},j}$) with age is not modeled. After simplification we obtain:

10 $$ {\displaystyle \begin{array}{c}1- RM{C}_j^{\mathrm{p}}=\left(\frac{1}{H{R}_j^{\frac{1}{\propto }}}-1\right)\times RM{C}_j^{\mathrm{p}}\\ {}\iff RM{C}_j^{\mathrm{p}}=H{R}_j^{\frac{1}{\propto }}.\end{array}} $$

Equation 10 provides a striking result: when preventive care has the same effect on morbidity and mortality (and hazard *α* = 1), savings from avoiding morbidity equal life extension costs. When the relative impact of prevention is larger on morbidity than mortality (i.e., *RMC* < *HR*) and hazard *α* = 1, savings surpass life extension costs. This also holds for the case where the relative impact is the same but the hazard decreases with time (*α* < 1).

Based on eq. 10. an indicator for the ratio of savings from avoiding morbidity to costs during added life years is calculated as:

11 $$ \frac{1}{RMC_j^{\mathrm{p}}}\div \frac{1}{HR_j^{\frac{1}{\propto }}}. $$

If the indicator is larger than one, then savings surpass life extension costs.

### Consideration of discounting

Note that eq. 10, which refers to a single period *j*, overestimates life extension costs as the additional survival time needs to be discounted to present value. For the U.S. the recommended annual discount rate is 3% [13]. The model is able to consider discounting in the same way as the Declining Exponential Approximation of Life Expectancy (DEALE) method [14, 15] does. Based on the definition of the period-specific hazard ratio as ${\lambda}_j^{\mathrm{p}}/{\lambda}_j$ we obtain:

12 $$ {HR}_j=\frac{\lambda_j^{\mathrm{p}}+r}{\lambda_j+r}, $$

where *r* is the annual discount rate.

While *HR*_*j*_ is adjusted according to eq. 12, ${RMC}_j^{\mathrm{p}}$ (or savings) is not discounted as it refers to a single period *j* (eq. 10) and savings strictly occur during this period (whereas life extension costs are incurred beyond this period and therefore need to be discounted). Nevertheless, when considering a multi-period time horizon, the period of ‘normal’ life years (i.e., periods *j* = 2 to *n*) needs to be discounted in addition, as conventionally required.

### Calculation of cost-effectiveness

In case where savings from reducing morbidity are smaller than life extension costs, i.e., the intervention results in a net cost increase, the question appears whether this cost increase is justifiable in terms of health benefits (e.g., life years gained or quality-adjusted life years gained). Hence, an evaluation of cost-effectiveness is required. In the following, I present two ways of calculating cost-effectiveness, based on absolute and relative changes in costs and effects, respectively. The conventional approach to determine the incremental cost-effectiveness ratio (ICER) of prevention compared to no prevention divides the difference in costs by the difference in effectiveness or ∆*C*/∆*E*. The numerator can be subdivided as follows:

13 $$ ICER=\frac{-\Delta  MC+\Delta  LEC}{\Delta  E}.\kern0.5em $$

Note that a full assessment of cost-effectiveness also would need to consider program-related costs (as part of direct medical costs). Replacing the two cost components in the numerator by the terms in eqs. 2 and 8 yields:

14 $$ ICER=\frac{\left(-\sum \limits_{j=1}^n\left(\prod \limits_{i=1}^j{s}_i\right)\times \left(1-{d}_j\right)\times \left(1-{RMC}_j^{\mathrm{p}}\right)\times {C}_j\right)+\sum \limits_{j=1}^n\left(\prod \limits_{i=1}^j{s}_i\right)\times \left(\frac{1}{HR_j^{\frac{1}{\propto }}}-1\right)\times \left(1-{d}_j\right)\times {RMC}_j^{\mathrm{p}}\times {C}_j}{\Delta E} $$

Defining incremental effectiveness by life years gained, we obtain:

15 $$ ICER=\frac{\left(-\sum \limits_{j=1}^n\left(\prod \limits_{i=1}^j{s}_i\right)\times \left(1-{d}_j\right)\times \left(1-{RMC}_j^{\mathrm{p}}\right)\times {C}_j\right)+\sum \limits_{j=1}^n\left(\prod \limits_{i=1}^j{s}_i\right)\times \left(\frac{1}{HR_j^{\frac{1}{\propto }}}-1\right)\times \left(1-{d}_j\right)\times {RMC}_j^{\mathrm{p}}\times {C}_j}{\sum \limits_{j=1}^n\left(\frac{1}{HR_j^{\frac{1}{\propto }}}-1\right)\times \left(\prod \limits_{i=1}^j{s}_i\right)\times \left(1-{d}_j\right)} $$

Simplification of the numerator yields:

16 $$ ICER=\frac{\sum \limits_{j=1}^n\left(\frac{RMC_j^{\mathrm{p}}}{HR_j^{\frac{1}{\propto }}}-1\right)\times {C}_j\times \left(\prod \limits_{i=1}^j{s}_i\right)\times \left(1-{d}_j\right)}{\sum \limits_{j=1}^n\left(\frac{1}{HR_j^{\frac{1}{\propto }}}-1\right)\times \left(\prod \limits_{i=1}^j{s}_i\right)\times \left(1-{d}_j\right)}. $$

In line with eq. 12 the hazard ratio needs to be adjusted for the discount rate. As this adjustment only affects the life extension period itself, periods *j* = 2 to *n* (the period of ‘normal’ life years) need to be discounted in addition. This procedure does not result in double discounting.

An alternative and relatively easy way to comprehend the calculation of the cost-effectiveness of prevention is obtained by eliminating baseline costs and effects from eq. 16 and is based on relative changes in costs and effects:

17 $$ \varepsilon =\frac{\sum \limits_{j=1}^n\left(\frac{RMC_j^{\mathrm{p}}}{HR_j^{\frac{1}{\propto }}}-1\right)}{\sum \limits_{j=1}^n\left(\frac{1}{HR_j^{\frac{1}{\propto }}}-1\right)}. $$

Equation. 17 divides the relative change in costs over lifetime by the relative change in lifetime. Therefore, this ratio presents an elasticity (*ε*), which divides the percent change in one variable by the percent change in another variable. The elasticity is smaller than one because in the numerator a relative cost increase from a relative increase in lifetime (1/ ${HR}_j^{\frac{1}{\propto }}$) is reduced by relative period-specific costs of prevention compared to no prevention (${RMC}_j^{\mathrm{p}}$). Still, using this measure of cost-effectiveness requires an additional investigation into the willingness-to-pay threshold, which is different than that for the ICER.

## Age-dependence of costs and cost-effectiveness

The cost impact of varying the age of prevention onset depends on changes in the relative costs and the hazard ratio of mortality of prevention. If the ratio of relative costs to the hazard ratio is constant independent of age, the ratio of savings from avoiding morbidity to life extension costs is, ceteris paribus, constant too (based on eq. 11). The absolute cost difference, however, increases, the lower the age of prevention onset: according to eqs. 2 and 8, increasing the number of time periods (consistent with an earlier age of prevention onset) increases any difference between prevention’s relative impact on costs and hazard rate of mortality in absolute terms. In case where savings from reducing morbidity are smaller than life extension costs, an increase in net cost from lower age of prevention onset needs to be balanced against an increase in health benefits.

## Application example

In the following, I present an application example of the model based on obesity prevention. As stated in the introduction, the theoretical model can be used both to determine cost-effectiveness as a primary source and to check the internal validity of other, more comprehensive models. First, I use the model to determine cost-effectiveness as a primary source. An exponential survival function with BMI as a control variable was recently used to model the probability of death for individuals with and without diabetes [16, 17]. This supports the use of an AFT model with a shape parameter of 1 to model survival in obese persons. A meta-analysis based on person-level data from 26 observational studies showed that the relative risk of death among individuals who are obese is 1.21 when compared with normal-weight people [10]. The relative risk decreases with age, being statistically insignificant at age 65 years and above [10]. Furthermore, adults (age ≥ 18 years) who are obese spend 42% more for medical care than normal weight people do [18].

Given that for obese persons the relative risk of morbidity (as approximated by expenditures) is larger than the relative risk of death, I predict based on eq. 11 that savings from avoiding obesity-related morbidity are also larger than costs during added life years. The savings potential of obesity prevention - measured in terms of excess spending due to obesity after considering the remaining lifetime - is approximately 17% (1.42/1.21–1). That is, consideration of life extension costs reduces the savings potential of obesity prevention measured as excess spending due to obesity from 42% (solely based on the morbidity of obesity) to 17%. Based on the age-dependence of the relative risk of death, this result, strictly speaking, only applies to prevention below the age of 65. As a word of caution, consideration of discounting would reduce the amount of life extension costs somewhat. This would support the expectation of overall savings from obesity prevention.

The model also helps to validate the results of more complex cost-effectiveness models. Again, I focus on the example of obesity prevention. The model by van Baal et al. [9] calculates age-specific relative costs and mortality of obese compared to “healthy living” individuals. As shown in Table 1, at each age interval the relative mortality increase is larger than the relative increase in expenditures. Based on the model presented in this paper this finding translates into an overall expenditure increase of preventing obesity and thus confirms the result by van Baal et al. [9]. This result shows little sensitivity with regard to changes of the value of the shape parameter: A threshold sensitivity analysis shows that α must be larger than 3.2 in order for results to turn from a cost increase to savings.

**Table 1:** Results of the cost-effectiveness model by van Baal et al. [6]

Age group	Relative mortality increase of obesity (%)	Relative expenditure increase of obesity (%)
20–29 y	53–58	< 5
30–39 y	59–79	4–7
40–49 y	82–90	7–10
50–59 y	81–91	9–13
60–69 y	57–80	13–14
70–79 y	36–55	10–13

## Discussion

This work provides new insight into the lifetime cost consequences of prevention. As shown, under a Weibull AFT model the cost consequences of prevention are essentially driven by two factors: i) the relative reduction of morbidity-related costs, which determines the amount of savings from avoiding morbidity; and ii) the hazard ratio of death, which determines the amount of life extension costs. This finding may be surprising if one expects baseline spending and its potential for savings or future extension to be the major driver but it is important to realize that some canceling out of baseline costs occurs (see eq. 9). The model confirms the intuition that an intervention with high reduction in mortality and comparatively little reduction in morbidity has a high ratio of life extension costs to savings from morbidity reduction and therefore leads to a net cost increase. In order to reduce health expenditures, prevention thus needs to target diseases with low baseline mortality (which reduces the potential for mortality reduction) and high costs. Nevertheless, patients with high baseline mortality may still be prioritized over those with low baseline mortality on ethical grounds to avoid a double penalty.

The model can be used both for explanatory and predictive purposes. Hence, it can be used both to check plausibility of the results of other models and to conduct an independent analysis. Specifically, it could be applied as a ‘screening’ tool for net savings and recommend conventional decision modeling only in the case where net savings are not expected (as this increases the true probability of not being cost-effective). The ‘triage’ function of the model could be helpful in view of the limited number of researchers in the field.

An advantage of the model is that it does not rely on absolute cost data: Absolute cost differences between prevention and no prevention vary more with age than relative cost differences (*RR*_morbidity(p), *j*_) and thus are less generalizable across different age groups.

The analysis assumes that prolonging life leads to a proportional increase in survival costs based on the multiplicative relationship between factors 1/*HR* − 1 and ${C}_{\overline{\mathrm{p}}}$ in eq. 7. However, survival costs may increase less than proportional. The underlying reason is a postponement of the last year of life (LYOL), which becomes cheaper with age [19–21]. If parametrization were possible, one could accommodate these savings in the discount rate and hence *HR*_mortality(p), *j*_ based on eq. 12. Still, given that the main purpose of this model is explanatory, such accommodation is not needed simply for the fact that most analyses do not model LYOL costs. Note that when savings from postponing the LYOL are modeled by a reduction in mortality, savings from reduced morbidity still need to be accounted for separately. Although a reduction in mortality may be partially related to a reduction in morbidity, in a modelling exercise savings from reduced morbidity (which is a dynamic component as savings evolve over time) are not captured when projecting savings from postponing the LYOL (which is a static concept based on cross-sectional data on age of death and expenditure) [22]

When applied for predictive purposes, the model presupposes that a Weibull (AFT) or exponential survival function provides an adequate fit. Hence, before applying the model analysts need to check whether there is empirical evidence in support of a Weibull (AFT) or exponential survival model. This includes checking the model’s assumption that the shape parameter is constant over time. However, the shape parameter can vary over time, reflecting changes in the hazard: mortality rates increase exponentially with age, but the increase decelerates at late ages [23]. In order to accommodate changes in hazard over time, a piecewise Weibull model may provide a good fit. This model provides a very flexible framework for modeling survival data. Although it is, strictly speaking, a parametric model, it can approximate any shape of a nonparametric baseline hazard [24]. A piecewise model could also accommodate the age-specific changes in relative risks of morbidity and mortality as considered in eq. 9.

Still, a piecewise model requires the assumption of instantaneous jumps in the hazard rate. This assumption is avoided by poly-Weibull models [25, 26] in which the overall hazard is the sum of several independent components. In health care, the independent components can be modelled as competing risks of death [27]. Yet, in the absence of detailed information on causes of death, these models may encounter an identifiability problem whenever each of the hazard components is not sufficiently distinct given the data at hand [27]. But regardless of the availability of more flexible and perhaps better fit models, it may still be reasonable to use a Weibull (AFT) or exponential survival function to determine cost-effectiveness as a primary source either in a sensitivity analysis or as part of a Bayesian model averaging approach that weights various survival models according to their likelihood [28]. In addition, it could be used to check the internal validity of the model as part of a set of model checks [29]. Future research may investigate whether similar model simplifications are feasible with alternative survival model specifications, particularly with other distributions suitable for AFT models such as the log-logistic distribution. In addition, future research may adopt a societal perspective, which includes future non-medical costs, i.e., consumption net of production in added life years [30].

## Data Availability

Data sharing is not applicable to this article as no datasets were generated or analyzed during the current study.

## Footnotes

1 Note that the brief literature overview on obesity models simply serves to motivate this study. I do not claim completeness or systematic treatment of the subject.

2 The underlying assumption is that it is possible to decompose *t* into *n* periods, each with a standardized length of 1. It holds because $t\times \left(1/{HR}_j^{1/\upalpha}-1\right)=\sum \limits_{j=1}^n\frac{t}{n}\times \left(1/{HR}_j^{1/\upalpha}-1\right)$.
